# Mercury in European topsoils: Anthropogenic sources, stocks and fluxes

**DOI:** 10.1016/j.envres.2021.111556

**Published:** 2021-10

**Authors:** Panos Panagos, Martin Jiskra, Pasquale Borrelli, Leonidas Liakos, Cristiano Ballabio

**Affiliations:** a European Commission, Joint Research Centre (JRC), Ispra, Italy; b Environmental Geosciences, University of Basel, Switzerland; c Department of Earth and Environmental Sciences, University of Pavia, 27100, Pavia, Italy

**Keywords:** Sediment transport, Heavy metal, Soil contamination, Hg, Soil erosion

## Abstract

Mercury (Hg) is one of the most dangerous pollutants worldwide. In the European Union (EU), we recently estimated the Hg distribution in topsoil using 21,591 samples and a series of geo-physical inputs. In this manuscript, we investigate the impact of mining activities, chrol-alkali industries and other diffuse pollution sources as primary anthropogenic sources of Hg hotspots in the EU. Based on Hg measured soil samples, we modelled the Hg pool in EU topsoils, which totals about 44.8 Gg, with an average density of 103 g ha^−1^. As a following step, we coupled the estimated Hg stocks in topsoil with the pan-European assessment of soil loss due to water erosion and sediment distribution. In the European Union and UK, we estimated that about 43 Mg Hg yr^−1^ are displaced by water erosion and c. a. 6 Mg Hg yr^−1^ are transferred with sediments to river basins and eventually released to coastal Oceans. The Mediterranean Sea receives almost half (2.94 Mg yr^−1^) of the Hg fluxes to coastal oceans and it records the highest quantity of Hg sediments. This is the result of elevated soil Hg concentration and high erosion rates in the catchments draining into the Mediterranean Sea. This work contributes to new knowledge in support of the policy development in the EU on the Zero Pollution Action Plan and the Sustainable Development Goal (SDGs) 3.9 and 14.1, which both have as an objective to reduce soil pollution by 2030.

## Highlights

• Hg pool in EU topsoils totals about 44.8 Gg, with an average density of 103 g ha^−^^1^.
• Coupling Hg stocks in topsoil with soil losses and sediment distribution in Europe.
• Hg displaced by water erosion is c.a. 43 Mg yr^−^^1^ and c.a. 6 Mg Hg yr^−^^1^ reach the rivers.
• Agricultural lands contributes to more than 85% of Hg losses.
• The Mediterranean Sea receives almost half of the Hg fluxes followed by Black Sea.

## 1 Introduction

Mercury (Hg) is an element that has no essential biological function and it is a serious threat to human health (Järup, 2003). It is liquid at room temperature and is 13.6 times heavier than water (Gochfeld, 2003). Heavy metal accumulation in soils is the sum of inputs from parent material, atmospheric deposition, industrial contamination, fertilisation and other agrochemical activities minus the crop removal, losses from leaching and volatization (Wuana and Okieimen, 2011). Mercury is emitted into the atmosphere from natural and anthropogenic sources. In contrast with most of the other heavy metals, mercury and many of its compounds behave uniquely in the environment due to their volatility and capability for methylation. In addition, mercury is more persistent in soils compared to ocean, lakes (where it is ultimately sequestered in sediments) or other biomes (Tangahu et al., 2011).

Humans have used mercury for pesticides, fungicides, gold mining and processing and chemical industry (Reimann et al., 2014). The main sources of Hg anthropogenic emission are combustion (fossil fuels, municipal and medical waste, sewage sludge, crematories), high-temperature processes (smelting, cement and lime production), manufacturing/commercial, gold extraction, fluorescent lamps and mine spoils (Huse et al., 1999; Mukherjee et al., 2004). A recent review has also addressed the Hg emissions close to mining activities and chrol-industries (Gworek et al., 2020). The annual global anthropogenic Hg emissions amount to ~2000–2500 tonnes (Zhang et al., 2016) and outweigh natural emissions (~500 tonnes yr^−1^, mainly from rock weathering and volcanic eruptions) (Amos et al., 2015; Bagnato et al., 2015) by an order of magnitude (Futsaeter and Wilson, 2013). Approximately two thirds of the emitted Hg mercury returns to the terrestrial system through precipitation and dry deposition (Zhou et al., 2021). The Hg accumulation in soils is about 800,000 tonnes in mineral soils and 150,000 tonnes in organic soils. In the European Union, the mercury emissions are around 80 tonnes per year (<5% of the total global ones) with the coal sector contributing most of it (Xu et al., 2015).

Mercury compounds are toxic to humans and animals. For example, the increase of mercury in freshwater may cause significant uptake by fish. The mercury contaminated fish and shellfish consumption in Minamata bay (Japan) in the early 1950s was a tragic event. Therefore, populations with traditionally high dietary intake of food originating from fresh or marine environment have highest dietary exposure to mercury (Zahir et al., 2005). Since Minamata mercury contamination event, the toxic burden of anthropogenic mercury (Hg) pollution for human and ecosystem health is globally accepted by policymakers and resulted in the UNEP Minamata Convention (UN, 2019) which entered into force in August 2017. This international binding treaty aims to limit mercury pollution's significant health and environmental risks by addressing provisions for mining and waste management of products containing mercury (Mackey et al., 2014).

The objectives of this study are to a) investigate the anthropogenic sources of Hg contamination in EU topsoils; b) assess the Hg stocks in topsoils per country and catchment and c) couple the Hg stocks with soil loss by water erosion to estimate the eventual Hg fluxes with sediment transport. This paper used the LUCAS survey to better understand the mercury concentrations in European Union and explain the hotspots concentrations comparing our results with existing literature. Finally, we make some considerations relevant to recent policy developments in the EU legislation.

## 2 Methods and data inputs

### 2.1 LUCAS topsoil database

The Land Use/Land Cover Area Frame Survey (LUCAS) is a project to monitor land use and cover changes in the European Union. In 2009, LUCAS included a soil module to monitor the soil health in the European Union. In LUCAS, the collected samples comprise five topsoils (0–20 cm) subsamples per location that are mixed to form a single composite sample; approximately 500 g of soil are air-dried before transferred to the laboratory (Orgiazzi et al., 2018).

**Fig. 1:**
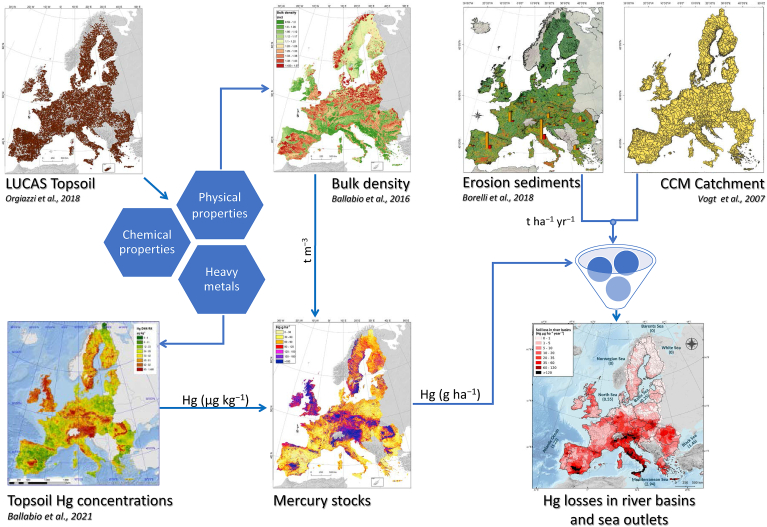
Data inputs and model integration for estimating Hg stocks and Hg sediment transport to river basins and sea outlets.

The LUCAS topsoil database has been compiled using the laboratory analysis of the physical properties (particle size distribution, coarse fragments, etc.), chemical properties (Nitrogen, Phosphorus, Potassium, etc.) and heavy metals (Fig. 1). Regarding heavy metals, the LUCAS topsoil database includes data for arsenic (As), cadmium (Cd), cobalt (Co), chromium (Cr), copper (Cu), iron (Fe), mercury (Hg), magnesium (Mg), manganese (Mn), nickel (Ni), lead (Pb), antimony (Sb) and zinc (Zn).

After the quality check, the LUCAS topsoil database 2009 includes 21,682 records (Fig. 1 – top left) and has been used in recent studies for heavy metals (Ballabio et al, 2018, 2021, 2018; Panagos et al., 2018). The 90 samples from Cyprus have not been analysed for mercury, and one sample was labelled with Hg concentration ‘Too high’ (excluded from our analysis as this sample had relatively high concentrations of nearly all metals). Therefore, for this study we will use the 21,591 records of the LUCAS topsoil database for the European Union countries and the UK.

### 2.2 Laboratory analysis of soil samples

In the first phase, LUCAS topsoil samples were analysed for soil physical and chemical properties (e.g. pH and texture) using various ISO methods. At a later stage, an ad hoc standard protocol was developed for heavy metals analysis. The LUCAS topsoil samples were subjected to the ISO 11466: 1995 method (ISO 11466, 1995) using aqua regia as the extracting agent of trace elements combined with microwave-assisted acid digestion (Nemati et al., 2010). Soil samples were treated by microwave assisted digestion followed by metal analysis by Inductively Coupled Plasma – Optical Emission Spectrometry (ICP-OES) as described in detail in Cristache et al. (2014). The detection limit for mercury is relatively low at 0.054 μg kg^−1^ and only 30 samples were found below this threshold. For those 30 samples, we have assigned the value of 0.027 μg kg^−1^ as a mean between 0 and the lower detection limit. We also applied quality control procedures throughout both the LUCAS soil survey and the laboratory analysis (Orgiazzi et al., 2018).

### 2.3 Mercury thresholds in soils

At European Union level, there is no common agreement on mercury threshold values for risk definition. Since the mobility and availability of mercury (and metals in general) depend on soil characteristics, such as organic carbon content, pH, texture and climatic conditions, guidelines and threshold values have been established as functions of these soil properties which differ from country to country (Carlon et al., 2007).

The ‘Predicted No Effect Concentrations (PNEC)’ value set by ‘*Registration, Evaluation, Authorisation and Restriction of Chemicals (REACH)’* regulation is about 22 μg kg^−1^ (ECHA, 2018). However, the screening values for intermediate (warning) risk from mercury (Hg) are highly diverse in the EU Member States varying from 500 μg kg^−1^ in Finland, 1000 μg kg^−1^ in Denmark, 2000 μg kg^−1^ in Austria and Slovakia and 20,000 μg kg^−1^ in Germany (Carlon et al., 2007).

The most cited guideline and threshold values for Hg have been proposed by the Finnish and Swedish legislations for soil contamination (Finnish and Swedish Ministry, 2007). According to the Government Decree on the Assessment of Soil Contamination and Remediation Needs 214/2007 (Finnish and Swedish Ministry, 2007), the Hg threshold value is 500 μg kg^−1^ and the lower and higher guideline values are 2000 and 5000 μg kg^−1^, respectively. Other authors have also proposed alternative indexes for defining the threshold such as the ratio of Hg to soil organic matter (de Vries et al., 2007).

### 2.4 Methods for estimating the Hg stocks and fluxes to river basins

The mercury content in topsoils was recently mapped at high resolution (Ballabio et al., 2021) highlighting the main drivers of mercury contamination in topsoils as well as the influence of soil organic matter, temperature, land cover and NDVI on Hg accumulation. This is the latest state of the art in mercury concentration in soils at European scale and has advanced both in the number of input samples compared to past assessments (Lado et al., 2008; Reimann et al., 2018) and in the machine learning techniques.

In the first step, we estimated the Hg stocks at pixel level (100 m spatial resolution) by multiplying the Hg topsoil concentration (0–20 cm) (Ballabio et al., 2021) with the topsoil bulk density (Ballabio et al., 2016) and the volume of soil for a depth of 20 cm (Eq. (1)).

[eq.1] Hg_stock_ (mg) = Hg_concentration_ (mg Mg^−1^) x Bulk Density (Mg m^−3^) x Volume (m^3^)

In 1 ha (ha) of 20 cm topsoil, the volume is 2000 m^3^. Depending on the bulk density (range: 1–1.4 Mg m^−3^; mean: 1.22 Mg m^−3^), the total weight of 1 ha topsoil has a range of 2000–2800 Mg. Therefore, the Hg stock depends on both the mercury content and the bulk density of the topsoils (Fig. 1).

In the next step, we combined spatially explicit estimates of hillslope-riverine system sediment fluxes with the Hg stocks to compute the amount of Hg potentially displaced together with soil particles; therefore drained into the nearest river. The hillslope-riverine system sediment and Hg fluxes are quantified on the basis of Borrelli's et al. (2018) quantitative estimates of soil erosion and deposition rates at EU scale. An assessment carried out using the RUSLE2015 dataset (Panagos et al., 2015), a high-resolution Digital Elevation Model (DEM) (25 × 25 m) and the spatially distributed sediment delivery model WaTEM/SEDEM (Van Oost et al., 2000).

According to Borrelli et al. (2018), the total soil displaced annually due to water erosion in EU is about 1 Pg yr^−1^ (10^9^ tonnes), of which about 0.16 Pg yr^−1^ pours into the riverine system. More than 93% of the sediment losses takes place in agricultural lands while forests have an overall surplus of sediments. The amount of mercury transported to EU rivers (Fig. 1) is provided using the pan-European river and catchment database named CCM (Vogt et al., 2007). To cover the entire study area, we included 5485 catchments, which drain into five main (Mediterranean, Atlantic Ocean, Black, Baltic, and North Sea) and three smaller sea outlets (Norwegian, Barents, and White Sea) (Supplementary Material S1).

## 3 Results

### 3.1 Descriptive statistics

For the 21,591 samples of LUCAS, the mean Hg value is 51 μg kg^−1^ and the median is 23 μg kg^−1^ with a range of 0–15.2 × 10^6^ μg kg^−1^. As referred above, 30 samples had values below the detection limit and 26 samples had 0 value and one sample had an extreme outlier. More than ¾ of the soil samples have Hg concentration less than 50 μg kg^−1^. Only 163 samples (0.8% of the total) have Hg concentration higher than the threshold set in relevant publications (>500 μg kg^−1^). This threshold is close the top percentile in Hg concentration (>0.422 mg kg^−1^) which accounts for 209 hotspots (Ballabio et al., 2021).

**Fig. 2:**
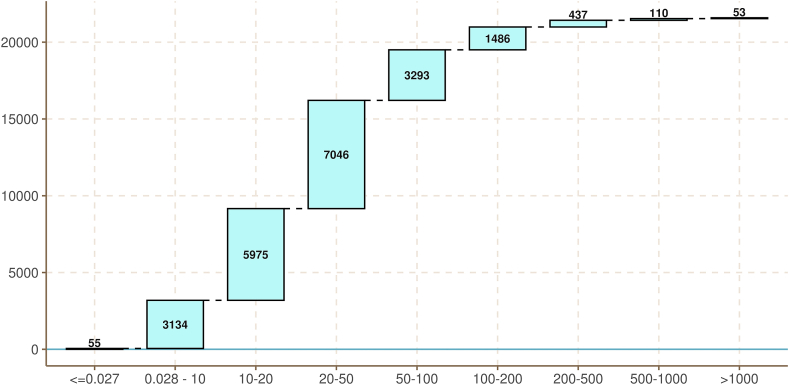
Number of samples per Hg concentration category.

We found 2086 records (9.6% of the total dataset) exceeding the threshold of 100 μg kg^−1^ (Fig. 2); that is commonly applied to distinguish between background (<100 μg kg^−1^) and Hg-enriched (>100 μg kg^−1^) sites (Gustin et al., 2008).

**Fig. 3:**
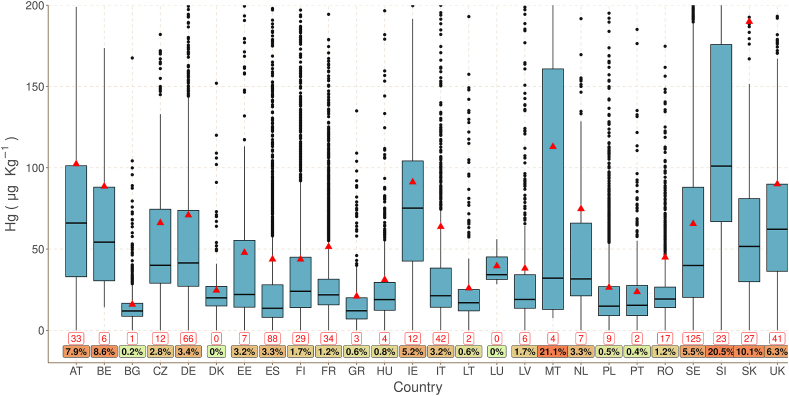
Mercury (Hg) concentration per country as μg·Kg^−1^. The horizontal line in the plot box is the median and red triangle is the mean Hg value. In the bottom, the red box is the number of samples with high Hg concentration >200 μg kg^−1^; below the proportion (%) of high concentration samples compared to the total number per country (scaled from green to red background). The boxplot is the interquartile range (IQR) expressed as the difference between the 25th (Q1) and 75th percentile (Q3); the bottom line is the result of the operation: Q1 - 1.5 * IQR and top line is the result of the operation: the Q3 + 1.5 * IQR. Dots outside the lines are considered as outliers. (For interpretation of the references to colour in this figure legend, the reader is referred to the Web version of this article.)

The highest mean values per country (Fig. 3) are in Slovenia (214 μg kg^−1^), Slovakia (190 μg kg^−1^), Malta (113 μg kg^−1^) and Austria (102 μg kg^−1^). The lowest mean values (all lower than 30 μg kg^−1^) are in Bulgaria, Greece, Spain, Poland, Portugal and Lithuania. The highest median is also in Slovenia (101 μg kg^−1^) followed by Ireland (75 μg kg^−1^) while Austria, United Kingdom, Belgium and Slovakia have medians in the range of 50–66 μg kg^−1^. Again, Bulgaria, Greece and Spain have very low median values (<14 μg kg^−1^).

On top of the statistical indicators (Mean, Median, Q1, Q3, IQR, etc.), we also compare the absolute number and the proportion of samples with high Hg concentrations (>200 μg kg^−1^) (Fig. 3). In such a comparison, Slovenia, Slovakia and Malta have at least 10% of their samples with concentrations >200 μg kg^−1^, followed by Belgium, Austria, UK and Ireland (all >5%). Furthermore, Hg concentrations are mapped per administrative unit at regional level to show spatial trends (Supplementary Material S2). However, it should be noted that soil is a continuous medium and administrative boundaries cannot influence the spatial distribution of chemical attributes or heavy metal concentration.

### 3.2 Mercury stocks in European topsoils

**Fig. 4:**
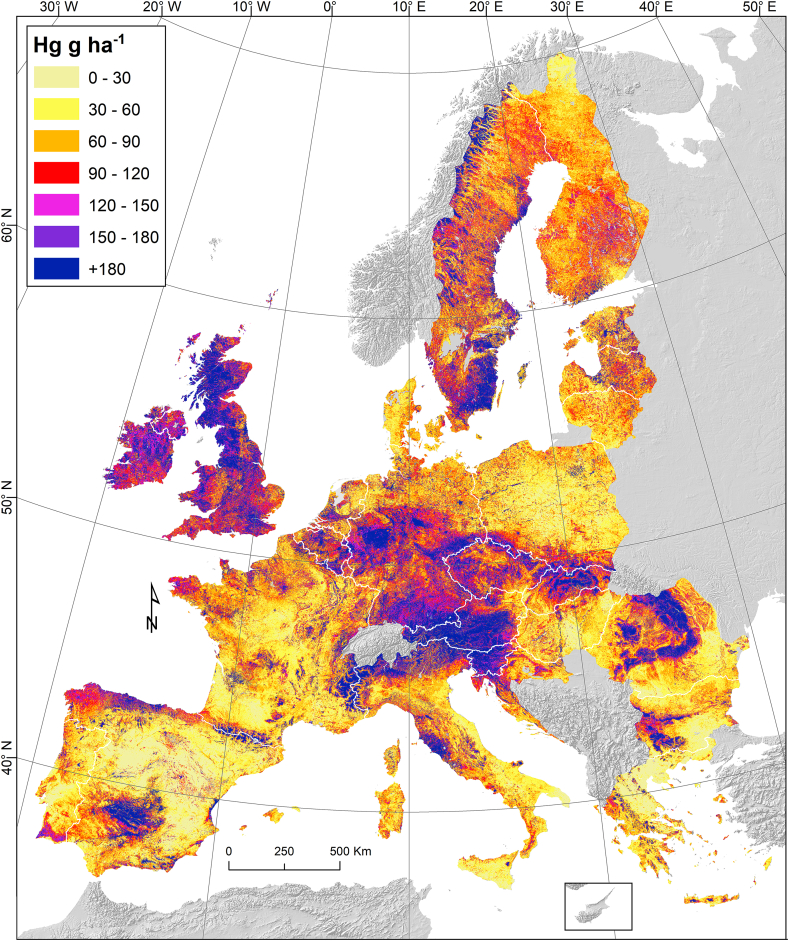
Map of Hg stock (g ha^−1^) in European topsoils.

Mercury stocks are very important for the global Hg community as they can be imported into regional and global Hg models. Today, best estimates of global Hg stocks in soils are associated with large uncertainties (Lim et al., 2020; Wang et al., 2019). The systematic sampling methodology and the amount of analysed samples in the LUCAS topsoil database (Orgiazzi et al., 2018) combined with an advanced machine learning model to estimate the Hg concentration (Ballabio et al., 2021) allow for a more precise estimation of the Hg stocks in European topsoils (Fig. 4). Compared to the Hg concentration map (Ballabio et al., 2021), the map of Hg stocks (Fig. 4) includes the variability of bulk density. Therefore, areas such as the Baltic States and Denmark with high bulk density (>1.25 t m^−3^) have relatively higher Hg stocks compared to their concentration (Fig. 4). For example, Lithuania and Greece have similar mean Hg concentrations but their differences in bulk density result in 35% variation in their Hg density. Even the spatial patterns between the Hg concentration map (Ballabio et al., 2021) and the Hg stocks (Fig. 4) are relatively similar, the bulk density introduces a difference between the two. In addition, the Hg stocks map is important as we need the mass in order to estimate the losses by water erosion.

**Fig. 5:**
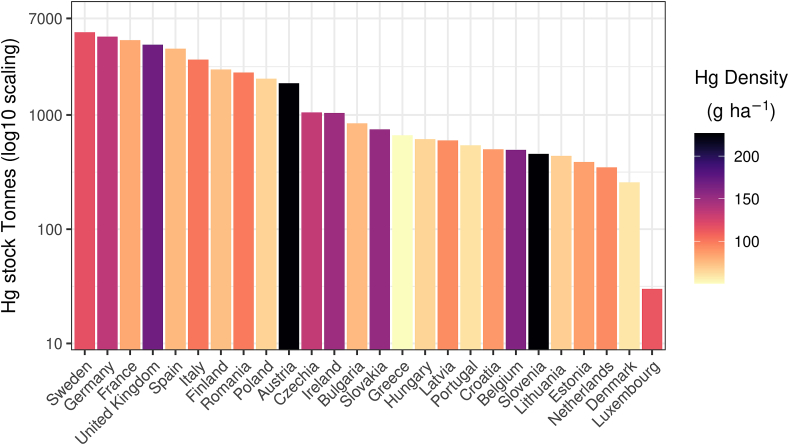
Hg stocks in Europe. Y-axis shows the Hg stocks (Tonnes or Mg) per country and the colour of the bar shows the Hg density (g ha^−1^). (For interpretation of the references to colour in this figure legend, the reader is referred to the Web version of this article.)

The total Hg stocks in the EU and UK topsoils is ca. 44.8 Gg. Bigger countries (e.g. Sweden, Germany, France, UK, Spain, and Italy) have the largest Hg stocks in topsoils (Fig. 5). In the contrast, in 16 Member States the Hg stocks are less than 1 Gg per country and the rest 10 countries store in total more than 37 Gg Hg in their topsoils.

The mean Hg density (as g ha^−1^) in the study area of EU and UK is about 103.2 g ha^−1^. Interesting to compare Slovakia which is almost 3 times smaller than Greece and has higher Hg stocks due to much higher Hg density (152 g ha^−1^ vs. 50 g ha^−1^). Similarly, Slovenia is more than 3 times smaller compared to Lithuania and has higher total Hg stock due to its Hg density (226 g ha^−1^ vs. 68 g ha^−1^).

### 3.3 Mercury losses with sediment fluxes

**Fig. 6:**
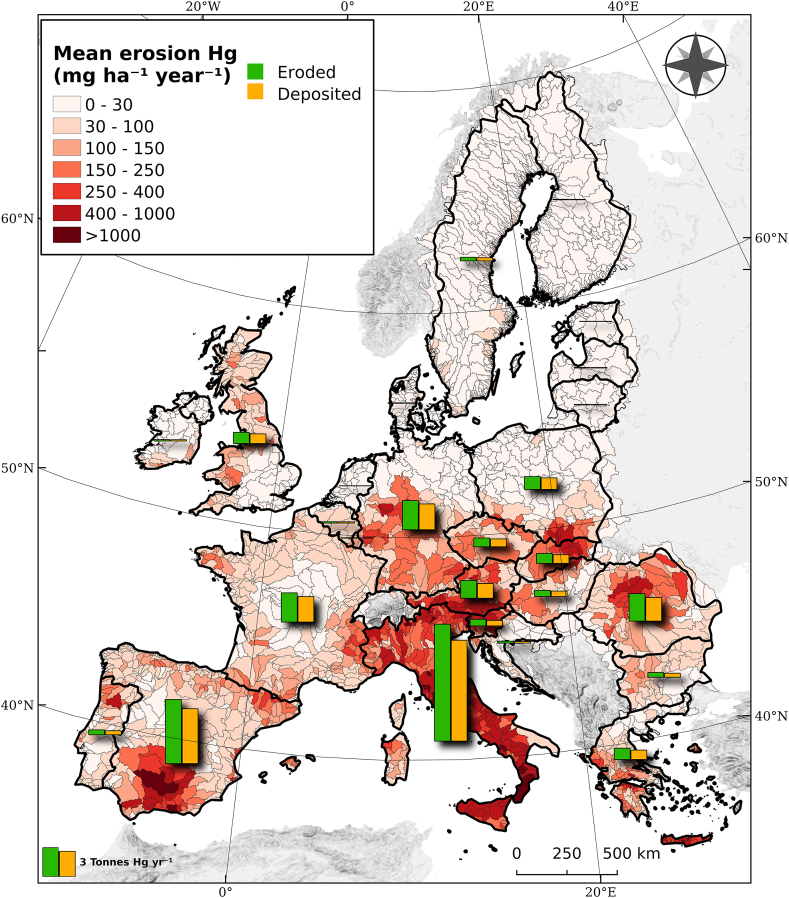
Estimated Hg displaced with water erosion per catchment. The vertical bars show the annual Hg eroded (green) and deposited (orange) per country. (For interpretation of the references to colour in this figure legend, the reader is referred to the Web version of this article.)

According to the sediment transfer dataset (Borrelli et al., 2018), the soil displaced due to water erosion accounts for less than 0.1% of the total topsoil in the field. Coupling the Hg stocks with the soil loss dataset (Fig. 1), we estimate the Hg displaced with water erosion to about 43.1 Mg yr^−1^. The Hg routed down from the hillslopes to the riverine system with the eroded sediments is about 5.9 Mg yr^−1^. The rest of the Hg (37.2 Mg yr^−1^) is re-deposited close to the eroded field. The mean Hg displaced with water erosion is c. a. 102 mg ha^−1^ yr^−1^ with major part of EU having very low rates (Fig. 6). The Hg losses with water erosion show a north-southwest (N-SW) oriented increasing gradient with Italy, south Spain, Slovenia, south Greece, south Austria and Slovenia having rates of Hg losses an order of magnitude higher than Scandinavia and northwest Europe. This depends both on the Hg stocks and the erosion rates which are very high in the Mediterranean basin. About 60% of the catchments (3200 basins) have Hg losses less than 30 mg ha^−1^ yr^−1^; most of them are in the Scandinavia and North Europe. In contrast, about 1% of the catchments have very high displaced Hg rates (>1,000 mg ha^−1^ yr^−1^) with Italy having 26 catchments with such peak losses (Spain 7, Greece 3 and Slovenia 2 catchments) (Fig. 6).

The vertical bars show the total eroded and deposited Hg (in tonnes yr^−1^) per country (Fig. 6). The amount of eroded Hg per country follows a much different distribution than the Hg stocks (Fig. 5) because of the soil erosion and sediment distribution rates. As an example, Italy has 4 times higher annual Hg losses compared to France even the Hg stocks in France are just 1.5 times higher the ones of Italy. Also, the Hg losses in Italy are c.a. 14.5 tonnes yr^−1^ and in Spain c.a. 8 tonnes yr^−1^ which sums more than half of the annual Hg losses in the EU and UK (Fig. 6).

**Fig. 7:**
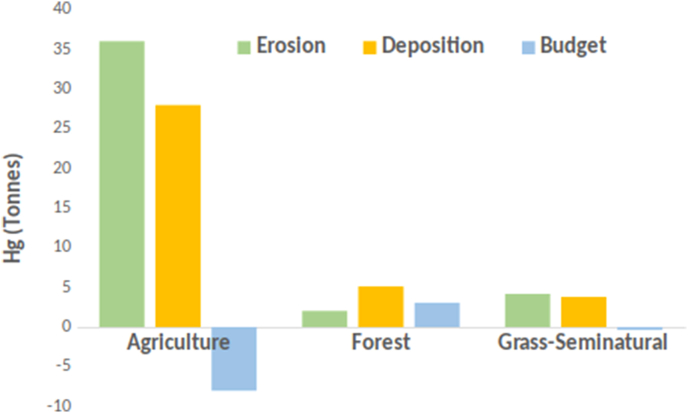
Annual Hg fluxes (gross erosion, deposition and budget) per land cover group.

Compared to forests and grasslands, the largest amount of soil losses to rivers occurs in agricultural lands (Borrelli et al., 2018). As gross losses, we consider the Hg displaced by water erosion and as net losses the Hg routed to the riverine system while the rest is deposited in the fields around. Therefore, the gross Hg losses due to water erosion in agricultural lands are 36.6 Mg yr^−1^, in forest 2.1 Mg yr^−1^ and in semi-natural areas 4.2 Mg yr^−1^ (Fig. 7). Thus, the net Hg losses (budget) due to water erosion from agricultural lands are about 8.2 Mg yr^−1^ while forests have a surplus of 3 Mg yr^−1^ due to sediment deposition (Fig. 7). Semi-natural areas and the rest of land uses have a relatively small total net Hg loss (0.6 Mg yr^−1^).

The deposition ratio is the fraction of Hg deposited in the catchment compared to the total Hg loss due to water erosion (Supplementary Material S3). The remaining fraction (1 minus deposition ratio) is the % of eroded Hg which is routed in the river basins. At EU level, the mean Hg deposition ratio is about 86% with the Mediterranean part having the lowest deposition rates.

**Fig. 8:**
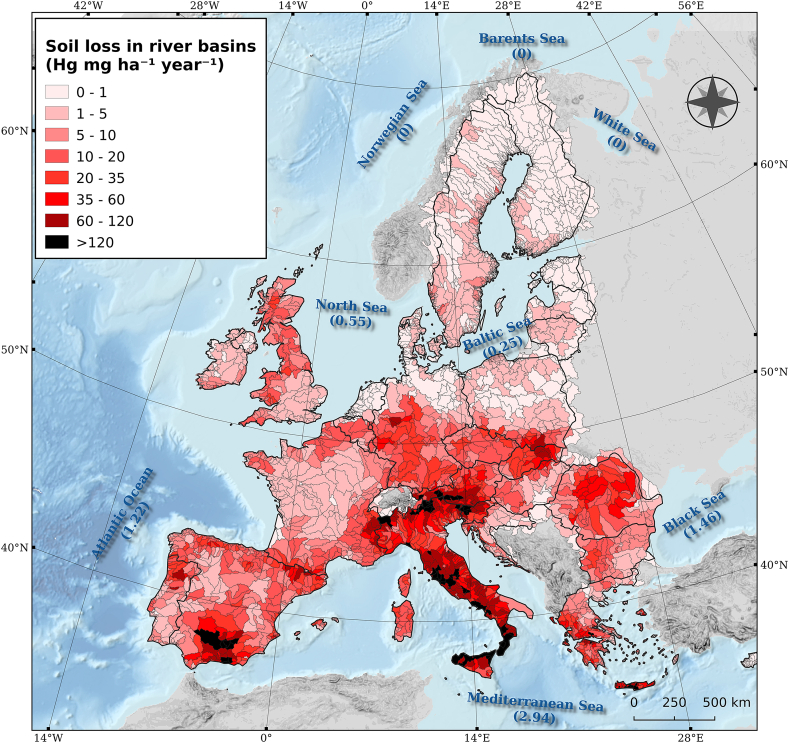
Estimated Hg losses to river basins and Hg fluxes to sea outlets (Mg yr^−1^) due to water erosion.

The catchments with high Hg stocks and high erosion rates have potentially the highest amount of Hg routed to the river basins (Fig. 8). In addition, the smaller the deposition rate, the higher Hg fluxes to sea outlets with sediments. The mean Hg loss in the river basins of the study area is about 14 mg ha^−1^ yr^−1^ with the Mediterranean basin having a mean of 32 mg ha^−1^ yr^−1^, Black Sea around 14 mg ha^−1^ yr^−1^ and the lowest value at the Baltic Sea (1.5 mg ha^−1^ yr^−1^). The big majority of the catchments (85.5%) have a mean Hg loss towards the riverine system of less than 5 mg ha^−1^ yr^−1^. Almost all of those catchments are in the Northern Europe and Scandinavia where soil erosion rates are very low. Conversely, only 79 catchments (1.5% of the total) have a mean Hg loss higher than 120 mg ha^−1^ yr^−1^; the major part of them are in Slovenia, Tuscany, Lazio, south Italy and Andalucía (Fig. 8). Out of the 244 catchments with Hg losses higher than 60 mg ha^−1^ yr^−1^, we found 76% of them in the Mediterranean area.

We also took into account the major sea outlets (Vogt et al., 2007) (Supplementary Material S1) that each catchment is linked to in order to estimate the seas with the highest “pressure” of Hg sediments. As Norwegian, Barents and White sea have very few catchments intersecting with the EU territory, the Hg potential losses (from EU territory) are very limited in those three sea outlets (<0.1 Mg yr^−1^). Almost half of the total Hg losses in the river basins are routed to the Mediterranean Sea (2.94 Mg yr^−1^) due to high sediment rates and Hg concentration in Slovenia and Italy. The Mediterranean Sea outlet covers about 0.9 × 10^6^ km^2^ (16%) of the study area, contributes to c.a. 50% of Hg losses in the seas (EU level) and 40% of its area has an Hg loss more than 20 mg ha^−1^ yr^−1^ routed towards the sea.

The North Sea and the Baltic Sea outlets have low rates of Hg losses in the river basins. 99.5% of the river basins in the Baltic Sea and 95% of the river basins in North Sea have Hg losses of less than 20 mg ha^−1^ yr^−1^. The total Hg losses in river basins which have as a sea outlet the Black Sea are estimated to 1.45 Mg yr^−1^ and the ones in the Atlantic ocean about 1.22 Mg yr^−1^. The sum of Hg losses in the sea outlets is a little bit higher (c.a. 6.4 Mg yr^−1^) compared to the total Hg losses in the EU and UK as some catchments in EU boarders (e.g. Balkans, East Europe, Finland, Baltic States) are tangent to non-EU territories (Fig. 8).

## 4 Discussion

### 4.1 High Hg concentrations close to mining activity in Europe

The annual mercury world production is about 2000 tonnes with main Hg mining countries being outside Europe (China, Mexico, Kyrgyzstan, etc.) (Reichl et al., 2014). Compared to c. a. 7000 tonnes of Hg production in the 1990s, we notice a strong decline following an increased concern about the Hg toxicity (Alloway, 2013). In addition, the UN Minamata convention on mercury prohibits primary mercury mining after a transition period of 15 years. The global mined Hg during the last 5 centuries is estimated to about 922 × 10^3^ tonnes with European sites producing more than 57% of the total Hg (Hylander and Meili, 2003). As an example, the Hg mining production in Almaden (Spain) have contributed to almost 1/3 of the total global Hg production (Hylander and Meili, 2003).

Soils can potentially be contaminated close to mining activities (or abandoned mines) because of mine wastes and residuals after refining the extracted metals (Wang et al., 2012) or from elevated atmospheric deposition (Ferrara et al., 1998). In the EU, the highest Hg deposit is in Almaden district (Spain) with 250,000 tonnes, followed by Idrija mercury mine in Slovenia with 140,000 tonnes and then by Mt. Amiata in Tuscany region of Italy with 100,000 tonnes (Rimondi et al., 2015). Here, we present the literature findings relevant to the high Hg concentrations close to these three mining activities plus additional results from mining activities in Asturias (Spain) and Rudnany (Central Slovakia).

The Idrija mercury mine in south-western Slovenia has produced around 107,000 tonnes of Hg with 45,000 tonnes released to the environment (Gosar et al., 2006). The Hg concentration in soils is decreasing with distance from the Idrija mercury smelter as the median Hg concentration in 1 km distance is about 47 mg kg^−1^, at 2 km this is much lower (3.2 mg kg^−1^) while at 3 km distance the median is 1 mg kg^−1^ (Gosar et al., 2006). It should be noted that Hg concentration in the air and soil has been drastically decreased after the mine production stopped (Kotnik et al., 2005). Therefore, the main Hg pollution source was the mine production.

The Almaden district in Spain has been mined from more than 2000 years and presents high mercury concentrations (Millán et al., 2006). In Almaden district, Hg concentration has high variability depending on the distance to the mine. The mean Hg is 2.3 mg kg^−1^ in 20 km distance from the mine, while it reaches Hg concentration of 97 mg kg^−1^ in 1 km distance (Lindberg et al., 1979). In a about 200 km west to Almaden, in the southern of Badajoz province (close to the boarders of Portugal), an abandoned cinnabar mining area is an Hg hotspot (García-Sánchez et al., 2009). In Asturias (NW of Spain), more than 20 abandoned Hg mines contributed to high Hg concentrations in this area (4.1 mg kg^−1^) (Loredo et al., 2006).

The mineralised volcanic area of Monte Amiata (120 km northwest of Rome) in the Tuscan is one of the largest mercury deposits with a cumulative production of more than 100,000 tonnes of Hg taking place from 1870 to 1980 (Rimondi et al., 2015). The entire eastern site of Mt. Amiata is very rich in mercury with concentrations reaching 220 mg kg^−1^ (Ferrara, 1999). In central Slovakia, the Rudnany iron ore mine is the largest source of mercury emissions in the country (Maňkovská, 1996). Close to this mining site, the Hg concentrations in soils reach 130 mg kg^−1^ even if the mining activity has been interrupted in 1980 (Angelovicova and Fazekasova, 2014). Also, the mining and smelting district of Pribram (Czech Republic) has shown high Hg concentrations (>1 mg kg^−1^), especially in the forest areas (Ettler et al., 2007). However, higher Hg concentration in forest areas may involve Hg cycling in forest ecosystem.

Those mining areas (Almaden district, Asturias, Monte Amiata, Idrija, central Slovakia and district of Pribram) have been spotted as outliers in the recent Hg assessment in Europe (Ballabio et al., 2021) and have Hg concentrations which are orders of magnitude higher than the median EU (0.038 mg kg^−1^). Therefore, the Hg stocks close to those mining areas are higher than 300 g Hg ha^−1^. The combination of high Hg stocks with high erosion rates in the sites of Slovenia, Italy and Spain results in outliers of Hg losses in river basins (Fig. 8). Therefore, high soil losses by water erosion from Hg contaminated sites and their transport through sediment routes can be of paramount importance for Hg losses to aquatic systems.

### 4.2 Hg concentrations close to chlor-alkali plants

Historical mercury contamination of land and waterways from chlor-alkali plants is a big environmental problem at some sites. One of the main technologies applied for the chlorine production uses mercury and this is a potential source of high Hg concentrations (Mukherjee et al., 2004). Hg released from chlor-alkali plants can enter the aquatic systems, oxidise, bind easily to suspended particles, and is deposited in the bottom sediments (Gray et al., 2004). Thirty years ago, chrol-alkali industries used mercury as cathode for their products (Biester et al., 2002). The waste discharge of those industries has potentially polluted areas close to chrol-alakli industries as Biester (2002) reported values in the range of 4–6 mg kg^−1^ in the soils in 1 km distance from those industries. Compared to the rest of the world, chlorine production was extensively high in western Europe (Brinkmann et al., 2014).

In this paragraph, we list some literature findings of high mercury concentration close to chlor-alkali plants. The spatial distribution of choral alkali plants in areas such as the Northern shore of the lake Vanern (Lindeström, 2001) in south Sweden, the Rm Valcea region (Romania) (Bravo et al., 2010), the area close to Torrelavega chlor-alkali plant in Santander (Gluszcz et al., 2012) and hotspots close to Lyon (Brinkmann et al., 2014 as in Fig. 1.3) can explain the high Hg concentration. Other places having high Hg concentrations are the Thur valley (France) close to Strasbourg polluted in early 2000's by chlor-alkali industries (Hissler and Probst, 2006), the areas close to Amsterdam metropolitan area (Bernaus et al., 2006) and the Ems estuary polluted by chlor-alkali plant close to Delfzijl (North Germany) in early 1970's (Essink, 1980). Those are some additional sources of anthropogenic Hg contamination, which can be added to the past mining and coal combustion activities explaining the Hg outliers in Europe (Supplementary Material S4). Thus, we mapped the main 74 chlor-alkali plants using the recent literature reviews on the topic (Brinkmann et al., 2014; Gworek et al., 2020).

In addition, we made a GIS neighbour analysis to estimate the distance of chlor-alkali plants to the Hg hotspots (>422 μg kg^−1^) (Supplementary Material S4). We found that 13 Chrol-alkali plants are in a proximity distance of less than 20 km from the Hg hotspots. Those 13 explained hotspots plus the mining-smelting district of Pribram are added to the 87 explained hotspots (Ballabio et al., 2021) reaching almost 50% (101 hotspots) of the 209 Hg hotspots in LUCAS topsoil database (>422 μg kg^−1^). Therefore, our study contributes to the global inventory of Hg hotspots which estimates around 3000 polluted sites worldwide (Kocman et al., 2013).

The process improvement in chlor-alkali industries (conversion to the membrane process not involving Hg) has decreased the mercury waste about two orders of magnitude after the 1980s (Pacyna et al., 2001). In relation to soils, the positive effects of this process improvement can be observed long after the conversion.

### 4.3 Urban centres and local Hg diffuse contamination

According to Geochemical Mapping of Agricultural and Grazing Land Soil in Europe (GEMAS) database, several cities (Dublin, London, Paris, Rotterdam, Rome) have shown some high Hg concentrations (Reimann et al., 2014). Those anomalies prove the impact of “old” chemical industry production close to urban centres, hospitals, waste incinerators and crematoria (Ottesen et al., 2013). According to our study, we found Hg anomalies close to urban centres such as Liverpool, London, Paris, Madrid, Craiova, Amsterdam, Milano, Civitavecchia (port of Rome). Similar findings about Hg anomalies close to big cities due to urban agglomerations have also reported for London, Rotterdam and Paris by another studies (Baize et al., 2001; Ottesen et al., 2013).

In addition to mining activities, coal combustion and chlor-alkali industries, the mercury contamination much depends from past or present local diffuse pollution activities such as small-scale industries employing mercury (scientific instruments, electrical equipment, dental amalgams, felt making, disinfectants, and production of caustic soda). In Oost-Vlaanderen region (Belgium), the industrialised areas around Lokeren, Hamme and Kruibeke which are close to felt production where mercuric nitrate was extensively used, showed high mercury concentrations (>1000 μg kg^−1^) (Tack et al., 2005).

### 4.4 Mercury fluxes to sea outlets

The main concern of Hg exposure to human is through the transfer of Hg from soils to aquatic ecosystems. The amount of mercury that enters into the aquatic environments due to erosion and riverine transport is unknown and hard to establish due to the lack of suitable data (Kocman et al., 2013). Here, we combined spatial datasets of water erosion, sediment transfer and Hg stocks to model the annual Hg fluxes in aquatic systems at continental scale. For EU and UK, the Hg losses to sea outlets due to water erosion is about 6 Mg yr^−1^ (Fig. 8).

In aquatic ecosystems, Hg can be methylated to Methyl-mercury, which is highly toxic and is accumulated by the biota and biomagnified though the food chain. The mercury concentrations in sediments influences the Hg level in water reservoirs or seas close to contaminated areas industries (Gworek et al., 2016). Mercury concentration in the oceans and seas depends on many factors such as atmospheric deposition, sediment transport, land degradation, local contamination, etc. (Gworek et al., 2016). It is estimated that the riverine fluxes in the Mediterranean Sea are about 26 nmol Hg m^−2^ yr^−1^ (eq. 50 mg yr^−1^ ha^−1^) which are half compared to the ones in South China Sea (Lamborg et al., 2014). The main reason for this difference is either the lower concentration of Hg in sediments of the Mediterranean basin or the distance from the sea of Hg hotspots. With the exception of Gulf of Trieste and the Tuscany coast (archipelago Toscano), most of the EU hotspots are not close to the seashore. The Gulf of Trieste is subject to substantial Hg pollution as it is not far from the Slovenian hotspots of Idrija. This part of EU is among the most susceptible to soil erosion and sediment transport (Borrelli et al., 2018); therefore, mercury is drained from soil of the Slovenian mines and transported to the Gulf of Trieste (Žagar et al., 2006). Few heavy-storm rainfall events during autumn trigger more than 85% of the erosion in the area (Panagos et al., 2016) and the mercury transport to the Gulf of Trieste.

In this study, we modelled the impact of water erosion (soil loss due to rill and sheet erosion) on sediment distribution and the Hg losses in the river basin. However, other soil loss processes such as gully erosion, landslides or wind erosion are not considered due to lack of spatial distributed data. In addition, the Hg losses presented here are long-term averages and cannot be compared with specific annual point losses. In the Mediterranean basin, the total Hg losses with sediment transfer is estimated at 8 Mg yr^−1^ (Rajar et al., 2007) which is about 2.5 times higher than our estimate. The reasons for this difference are: a) we include only the basins originated from catchments in the EU and we do not take into account sediment losses from western Balkan countries and North Africa; b) gully erosion and landslides are processes which have an important contribution to sediments (even larger than sheet and rill erosion) in the Mediterranean (Poesen, 2018) and c) soil loss rates have decreased by 19% in agricultural lands (Panagos et al., 2015) during the last 15 years as the two studies focus on different periods. As soon as soil losses from gullies and landslides are quantified, it will be worthy modelling the sediment fluxes from areas where those erosion processes are dominant.

For the Black sea, the river basin sediments are the major source of mercury in the aquatic system. The Hg total river inputs to the Black Sea are about 24.5 kmol yr^−1^ (eq. 4.9 Mg yr^−1^) (Rosati et al., 2018) which is 3 times higher compared to the 1.46 Mg yr^−1^ of this study. This difference is justified as we modelled the Hg losses from EU Catchments (mainly Danube). Hg losses from non-EU catchments (from Russia, Ukraine, Turkey, etc) are not included in our study compared to the one of Rosati et al. (2018).

The future projections of mercury losses are positive as it is expected a decrease due to specific control technologies and legal biding regulations (Krabbenhoft and Sunderland, 2013). In contrast, climate change projections estimate an increase in rainfall erosivity by 18% in EU in next 30–40 years (Panagos et al., 2017) or even more (Borrelli et al., 2020) rising the soil losses by water erosion and facilitating sediment transport. Therefore, the future estimates of Hg losses to seas depend on both expected decrease of Hg soil concentrations and projected increase of soil losses.

Both the Hg stocks and the Hg losses per catchment will be made available in the European Soil Data Centre (ESDAC). Making available all input datasets such as Hg concentrations, Hg stocks, bulk density, sediment fluxes, catchment soil losses and Hg fluxes due to erosion, we facilitate modelling advancements in this topic.

## 5 Policy and future actions

Policy actions at national, regional, and global scales have addressed mercury pollution sources. Globally, the Minamata Convention is a new legally binding international agreement designed to protect human health and the environment from anthropogenic emissions and releases of mercury. Experience with regional mercury management suggests that future policy should take into account transboundary influences, coordinate across environmental media, and better assess human and ecological impacts in regulatory analyses (Lamborg et al., 2014; Obrist et al., 2018).

There is also a need to track and determine the degree to which Hg inputs to coastal waters are changing due to changes in watershed deposition. In 2005, the European Commission adopted a comprehensive plan to address mercury pollution (European Commission, 2005). The Mercury Strategy listed 20 actions to reduce mercury compounds in products and devices (e.g. thermometers, barometers), set new rules for sage storage of mercury and include provisions on mercury emissions in order to protect people against exposure. In 2012, the EU regulation (European Commission, 2012) recognised mercury and its compounds as highly toxic to humans, ecosystems and wildlife and phases out of the market a number of devices working with mercury.

Recently, the European Commission announced a very ambitious package for a non-toxic environment in the European Union within the EU Green Deal (Montanarella and Panagos, 2021). In May 2021, the European Commission adopted the *Zero Pollution Action Plan for water, air and soil* (ZPAP, 2021) to better prevent, remedy, monitor and report on pollution. One of the objectives of this policy development is to better monitor the current state of diffuse pollution in soils (e.g. heavy metals included) and to estimate the pollution in waters due to sediments. Therefore, this assessment contributes to establish baselines on mercury diffuse pollution and fluxes in EU.

At global scale, the United Nations has adopted the Sustainable Development Goals (SDGs) agenda with 17 main SDGs and a monitoring framework of 231 indicators. Among them, the SDG3.9 puts as an objective to substantially reduce the number of deaths and illnesses from hazardous chemicals and air, water and soil pollution and contamination by 2030. In relation to SDG3.9, the challenge is to reduce potential mercury poisoning. The SDG14.1 targets to reduce marine pollution of all kinds and the challenge here is to decrease the risk of mercury leakages into rivers and oceans (Hirons, 2020). This study may contribute to the development of indicators to estimate the progress both for the EU policy developments (Mercury regulation, Zero Pollution Action Plan) and the SDGs targets.

## 6 Conclusions

The LUCAS topsoil database, including the results of the 22,000 analysed soil samples, is a valuable input that enabled to estimate the Hg concentration in topsoils, the stocks, and the fluxes to river basins at continental scale. In addition, this is the first study to couple soil diffuse contamination of an emerging pollutant (mercury) with sediment distribution models at continental scale.

In this study, we estimated the Hg stocks in topsoils at 44.8 Gg with a mean density of 103 g ha^−1^. Then, we coupled the Hg stocks with the pan-European sediment distribution model outputs to estimate the Hg displaced annually by water erosion to at 43 Mg yr^−1^ (c.a 0.1% of the total Hg stocks). Agricultural lands contributes to more than 85% of those losses. As a follow-up, we used the European Catchment and River database to estimate the total Hg losses in river basins. In EU and UK, the total Hg losses in river basins is about 6 Mg yr^−1^ which is 14% of the total displaced Hg as the rest is re-distributed close to the eroded field. The catchments with high Hg concentration and high erosion rates are the ones with extreme Hg annual losses. Therefore, we estimated that 1.5% of the river basins have Hg losses higher 120 mg ha^−1^ yr^−1^; all of them are located in the Mediterranean Basin.

The delineation of river basins allows to estimate the Hg losses which potentially can reach the major European Sea outlets. Summing up the possible Hg losses to sea outlets, we conclude that the Mediterranean Sea gets 2.94 Mg Hg yr^−1^ which is half of the total Hg losses routed to EU river basins. The Black Sea get c.a. 1.46 Mg Hg yr^−1^ while the Baltic Sea gets only 0.25 Mg Hg yr^−1^ and North Sea around 0.55 Mg Hg yr^−1^. Those are the Hg losses attributed to water erosion in the EU and do not include gullies or landslides.

Taking into account the current policy developments at global scale with the Minamata Convention and the adoption of the Sustainable Development Goals (here we focus on SDG 3.9 and SDG14.1), this study offers some insights in the mercury stocks in EU topsoils and fluxes to the river basins and Sea outlets. In EU policy area, the adopted Zero Pollution Action Plan raises the issues of diffuse soil contamination in EU and envisages the development of relevant indicators to monitor the progress in soil pollution.

## Declaration of competing interest

The authors declare that they have no known competing financial interests or personal relationships that could have appeared to influence the work reported in this paper.
