# Supplementary material for: Mercury in European topsoils: Anthropogenic sources, stocks and fluxes
Source: Environ Res. 2021 Oct;201:111556. doi: 10.1016/j.envres.2021.111556 (PMC8503384; doi:10.1016/j.envres.2021.111556)
Supplement: Multimedia component 1 [file mmc1.docx]

**Supplement Material for the manuscript:**

**Mercury in European topsoils: anthropogenic sources, stocks and fluxes**


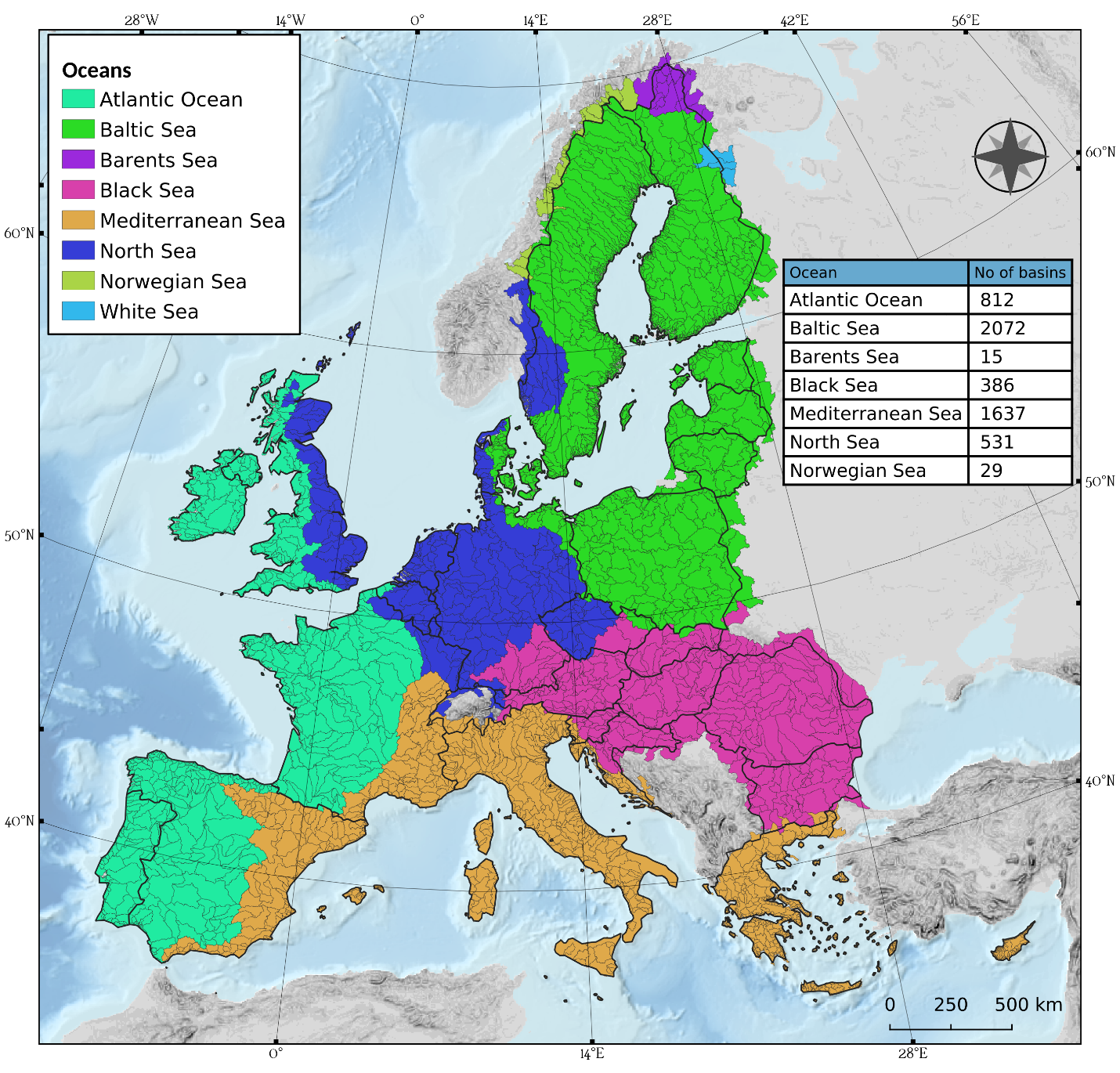


**Figure S1**: Delineation of the River basins and main Sea outlets in EU and UK.


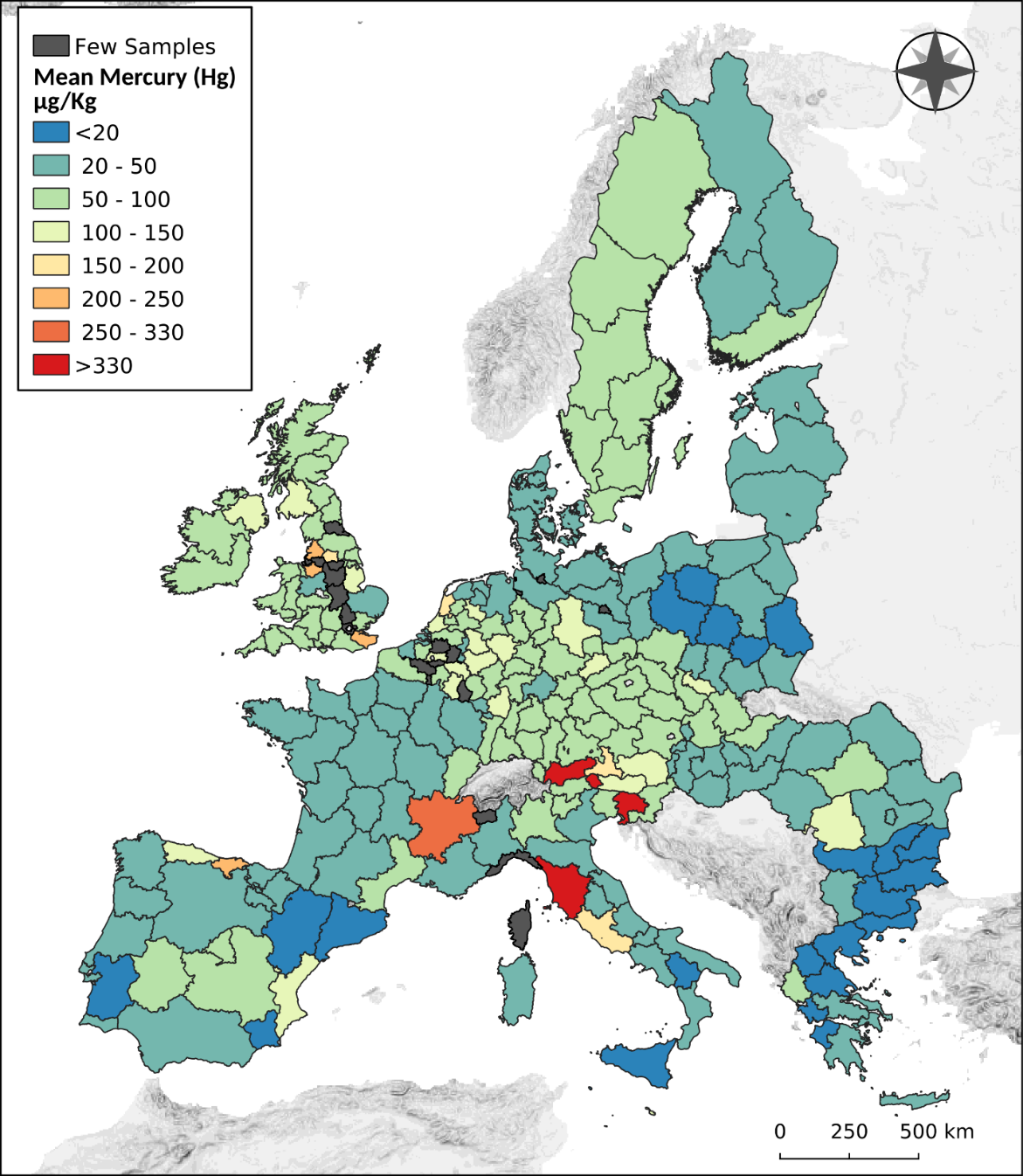


**Figure S2.** Mean Hg concentration (μg Kg^-1^) by NUTS2 region in the European Union and UK. Metropolitan cities and regions with few samples (< 5 samples) are masked out.


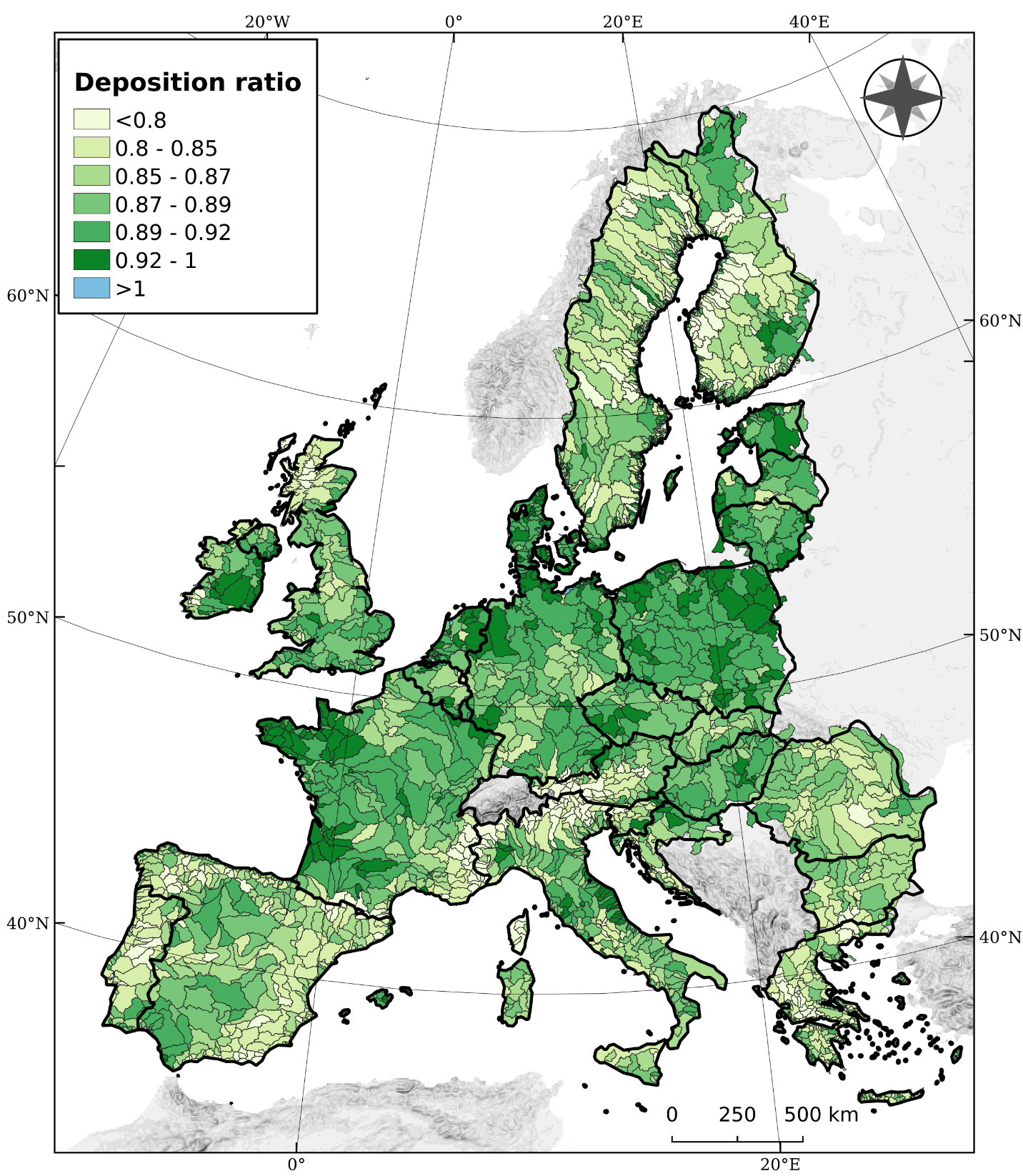


**Figure S3**: Deposition ratio of Hg losses.


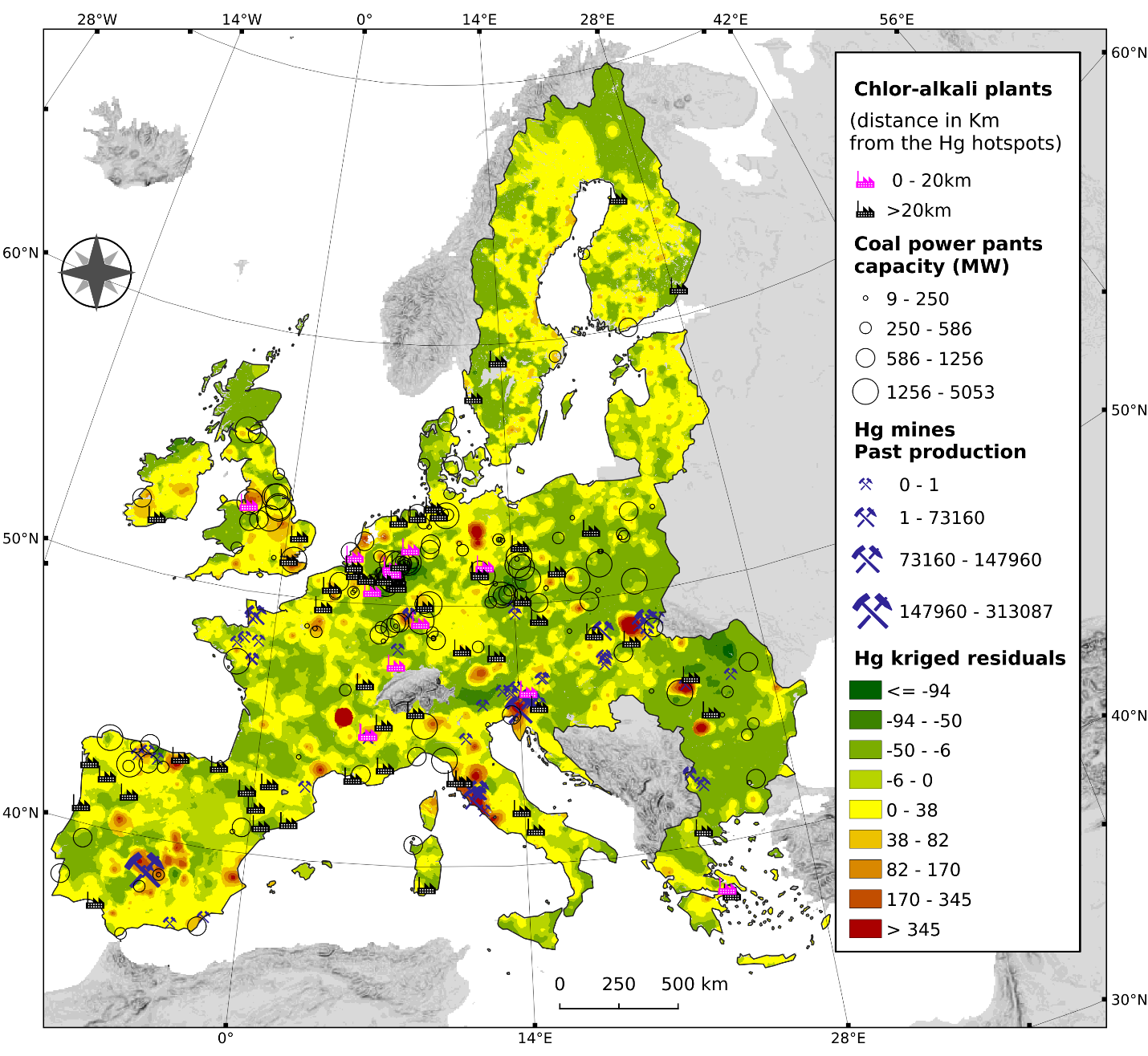


**Figure S4.** Map of kriged residuals including outliers overlaid with a) Chlor-alkali plants (in 2 categories based on their distance to hotspots); b) coal fired power plants in circles with the size of the circle proportional to the power production and c) Hg mines with the size of the symbols representing their past production (where available). Compared to the figure of Ballabio (2021), this one includes also the Chlor-alkali plants.
